# Microbial influences on HPV infection and cervical carcinogenesis: emerging evidence from the vaginal microbiome

**DOI:** 10.3389/fmicb.2025.1733315

**Published:** 2025-12-16

**Authors:** Xue Feng, Wei Song, Xinyue Ren, Zhihui Xu, Chaoyang Li, Min Feng, Nan Wang

**Affiliations:** 1 Department of Clinical Laboratory Medicine, The First Affiliated Hospital of Dalian Medical University, Dalian, Liaoning, China; 2 College of Laboratory Medicine, Dalian Medical University, Dalian, Liaoning, China; 3 Institute of Pulmonology, The First Affiliated Hospital of Dalian Medical University, Dalian, Liaoning, China

**Keywords:** cervical cancer, community state types, human papillomavirus, lactobacilli, vaginal microbiota

## Abstract

Microbial communities play a vital role in the human defense system, existing symbiotically with us, contributing to metabolic processes, and strengthening immune defenses against pathogens. A diverse bacterial population in the vagina contributes to maintaining dynamic homeostasis, with their interactions playing a critical role in determining health or disease status. The balanced vaginal microbiota, dominated by *Lactobacilli*, helps maintain vaginal pH, converts glycogen to lactic acid, and produces bacteriocins and hydrogen peroxide (H_2_O_2_), all contributing to its protective functions. On the other hand, an abnormal vaginal microbial composition, characterized by a decrease in beneficial microorganisms, heightens the risk of gynecological diseases such as bacterial vaginosis (BV), sexually transmitted infections, human papillomavirus (HPV) infections, and cervical cancer due to persistent infections. Variation in microbial composition is influenced by factors such as racial background, ethnicity, pregnancy, hormonal fluctuations, sexual behavior, personal hygiene practices, and various physiological conditions. This review aims to offer a detailed overview of the existing literature, focusing on the complex interplay between vaginal microbiota and gynecological conditions such as HPV infection. Our goal is to provide valuable insights that can inform future clinical strategies and interventions.

## 1 Introduction

The human microbiome comprises many microorganisms, spanning bacteria, archaea, protozoa, fungi, and viruses. Together, their collective genetic material forms the “microbiome,” and they inhabit various body regions in staggering numbers (Schirmer et al., 2018). Their colonization within the human body stems from intricate interactions with multiple facets of host physiology and metabolism, reflecting a finely tuned relationship. They aid in nutrient absorption and the development of the host’s immune system, playing a vital role in safeguarding the host against infections. In essence, human rely on microbes to maintain their health, while microbes, in turn, depend on the distinctive environment provided by the human body to thrive and coexist in a mutually beneficial relationship.

Based on findings from numerous recent studies utilizing microbiome and omics approaches, alongside data from diverse cohorts of women, it’s evident that the female genital tract harbors a distinct microbial community. Notably, the vaginal microbiome is typically dominated by *Lactobacilli* (Ravel et al., 2011), maintaining a low-pH environment that protects against pathogens (Miller et al., 2016). Disruption of this community-marked by reduced *Lactobacilli* abundance and increased microbial diversity-has been associated with inflammation and a heightened risk of persistent HPV infection, ultimately contributing to the development of cervical lesions. However, several knowledge gaps persist. The vaginal microbiota, a key component of the reproductive tract microbiota, is influenced by changes in the gut microbiota (Xu, Mageswaran, et al., 2025). Moreover, the causal relationships among dysbiosis, HPV persistence, and cervical carcinogenesis remain incompletely understood.

This review aims to clarify these concepts and highlight how microbial ecology contributes to HPV susceptibility and disease progression. By distinguishing key microbial niches and emphasizing unresolved mechanisms, the review provides conceptual clarification and points toward areas for future study.

## 2 Vaginal microbiota

### 2.1 Composition and ecological characteristics of the vaginal microbiota

The vaginal microbiota represents a dynamic and intricately balanced ecosystem, with continual shifts in composition and interactions among its microbial members. In healthy women, it typically comprises six major phyla: *Firmicutes*, *Bacteroidetes*, *Fusobacteria*, *Actinobacteria*, *Tenericutes* and *Proteobacteria* (Lee et al., 2013), with *Firmicutes* predominating due to the abundance of *Lactobacilli*. While the healthy vaginal environment is inherently variable, it is typically characterized by a *Lactobacilli*-rich community that maintains ecological stability and epithelial protection.

### 2.2 Protective roles of lactobacilli in vaginal homeostasis

In the vaginal flora of most women, *Lactobacilli* are usually dominant at the species level and are commonly found in the vaginal epithelium. *Lactobacilli* protect by several mechanisms: (1) preventing pathogenic bacteria from adhering to the vaginal epithelium. Free glycogen is produced as the vaginal epithelium proliferates, sheds, and reattaches, providing a source of nutrients and energy for the growth of *Lactobacilli*, which in turn helps prevent the attachment of harmful bacteria. (2) *Lactobacillus* metabolizes glycogen via anaerobic glycolysis to produce lactic acid, which helps maintain a low pH environment in the vagina. Both protonated forms, D-lactate and L-lactate, exhibit bactericidal properties (O'hanlon et al., 2011). Lactic acid can cause the breakdown of bacteria apart from *Lactobacilli*, acidifies cell membranes, disrupts intracellular functions, and increases the permeability of cell membranes to H_2_O_2_, diacetyls, and other compounds, thereby strengthening its antimicrobial properties within the vaginal environment (Alakomi et al., 2000). (3) It produces these metabolites including H_2_O_2_, reactive oxygen species (ROS), bacteriocins, bacteriocin-like antagonistic substances, and bioemulsifiers. These metabolic derivatives prevent the growth of harmful bacteria and break down the biofilms produced by these harmful bacteria (Lee et al., 2013; Molina et al., 2024; Boskey et al., 2001). (4) Certain strains of *Lactobacilli* attenuate the proinflammatory response triggered by Toll-like receptors (TLRs) and exert immunomodulatory effects, partially due to the presence of lactic acid. Collectively, these functions position *Lactobacilli* as the primary regulator of vaginal microbial homeostasis.

### 2.3 Community state types and microbial transitions

**Figure 1:**
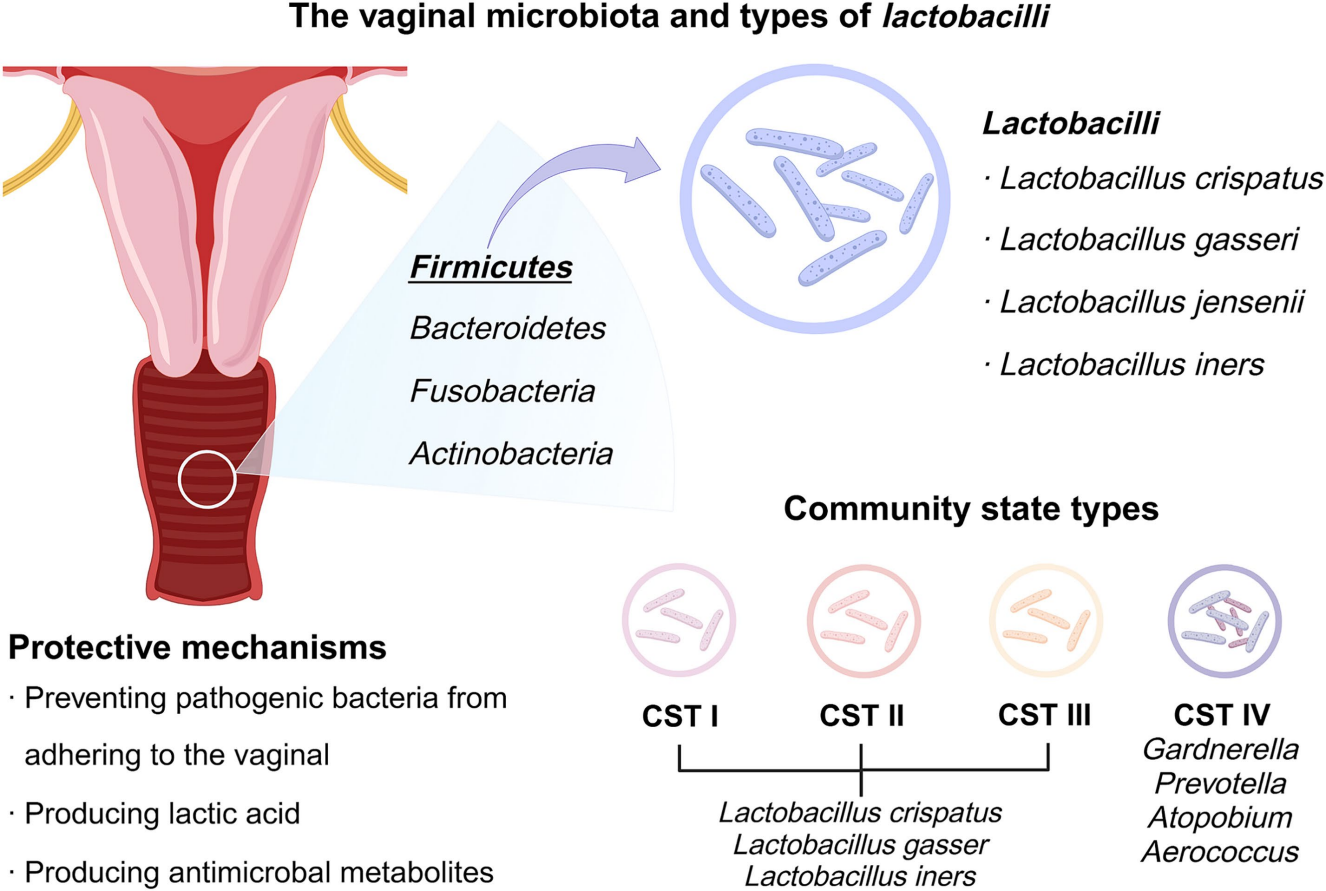
CSTs of the vaginal microbiota and the protective mechanisms of *Lactobacilli*. The figure shows the different CSTs of the vaginal microbiota and highlights how *Lactobacilli* contribute to vaginal health through mechanisms such as lactic acid production and pathogen inhibition.

According to species composition and the relative abundance of bacteria, the vaginal microbiota can be sorted into five distinct CSTs, initially introduced in 2011 (Ravel et al., 2011; Lee et al., 2020). These CSTs aid in research-driven interpretations of the health status, diversity, and transitional phases within the vaginal microbiome (Ravel et al., 2011). *Lactobacilli* (*Lactobacillus crispatus*, *Lactobacillus gasseri* and *Lactobacillus iners*) were the main features of CST I-III. In contrast, CST IV is a diverse group distinguished by a lower presence of *Lactobacilli*, typically with the highest pH median. The predominant bacteria in CST IV are anaerobes, including *Gardnerella*, *Prevotella*, *Atopobium*, and *Aerococcus*, which are frequently linked with BV (Figure 1). This cluster is labeled CST IV-BV, whereas another cluster lacking anaerobic bacteria is termed CST IV-AV (Di Paola et al., 2017; Brotman et al., 2014b). Both CST IV-AV and CST IV-BV are microbial communities characterized by reduced *Lactobacilli*, with CST IV-BV exhibiting even fewer *Lactobacilli*. In short, CST IV-AV is dominated by *Lactobacilli* and anaerobic bacteria, while CST IV-BV is characterized by BV-associated bacteria and high anaerobic diversity (Shen-Gunther et al., 2025). Conversely, vaginal dysbiosis is marked by reduced levels of *Lactobacilli* and elevated levels of CST IV microbiota (Auriemma et al., 2021).

### 2.4 Vaginal pH and its role in microbial and mucosal stability

The composition of the vaginal microbiota is closely intertwined with the vaginal pH. The indigenous flora is critical to the balance of the local microbiota, with a specific average pH for the entire biological response chain of 4–4.5. A vaginal pH below 4.5 provides a protective shield against pathogens, which struggle to thrive in such a low pH condition. When the pH level increases, it provides an opportunity for harmful bacteria to enter and disturb the balance of the vaginal environment (Borgogna et al., 2020; Mangieri et al., 2023). Thus, local homeostatic disorders and any pH alteration directly impair the integrity of the mucosa, which has a functional role in the internal and external adaptable slope (Borgogna et al., 2020; Mangieri et al., 2023). If ever disrupted, mucous membranes and tissues turn susceptible to invasive microbes and the process of inflammation. As a result, this hastens the degradation of the endothelial wall and the protective function of the microbiota, leading to increased accumulation of pro-inflammatory endotoxins and the prolonged spread of pathogens (Borgogna et al., 2020; Mangieri et al., 2023).

### 2.5 Determinants of vaginal microbiota composition

Various factors contribute to shaping the composition of the vaginal microbiota, such as race (Borgdorff et al., 2017), hormone levels (Krog et al., 2022; Adapen et al., 2022; María Fosch et al., 2022; Clabaut et al., 2021; Kaur et al., 2020), smoking history, vaginal douching practices, sexual activity frequency, persistent stress, and indiscriminate antibiotic use. In a cross-sectional survey comprising 396 women who are sexually active among four self-reported racial backgrounds, it was observed that the vaginal microbiota was not predominantly dominated by *Lactobacilli* among black and Hispanic women, a pattern considered normal and prevalent within these populations. These findings imply that genetic factors could play a notable role in shaping the makeup of the vaginal microbiota (Ravel et al., 2011). The vaginal microbiota has been shown to undergo cyclical fluctuations corresponding to different phases of the menstrual cycle (Chen et al., 2017). Additionally, the administration of external hormones, either orally or via injections, can alter its composition (Balle et al., 2020). Research demonstrated that following menopause, the percentage of indigenous bacteria, such as *Lactobacilli* declines (Lee et al., 2013). Furthermore, evidence indicates that individuals who smoke exhibit reduced levels of *Lactobacilli* (Brotman et al., 2014a).

## 3 Dysbiosis of vaginal flora

**Figure 2:**
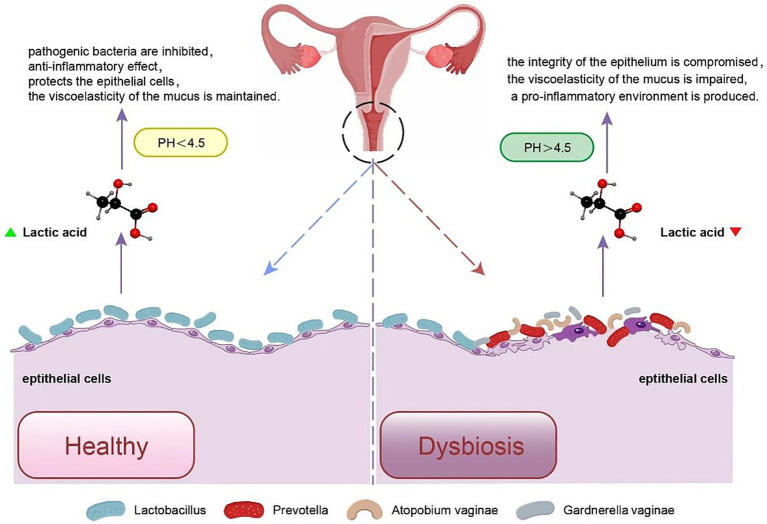
Female genital tract microbiota in health and disease. This illustration depicts the microbial composition and equilibrium of the female genital tract in healthy states, and shows how disruptions to this balance are linked to disease.

In the typical intestinal microenvironment, an increase in microbiome diversity is a sign of health, while the opposite trend is observed in the female vagina. The vaginal microbiota is a central component of vaginal microbiological ecology. Vaginal dysbiosis is distinguished as a decrease in the number of *Lactobacilli* and an increase in anaerobic bacteria, resulting in later metabolic and immunological alterations (Mckenzie et al., 2021). Typically, the components of vaginal microecology uphold a dynamic equilibrium. However, when the dominance of *Lactobacilli* is disturbed and microbial variety escalates, it initiates a sequence of metabolic alterations. Normal vaginal flora and dysbiosis are shown in Figure 2. The resulting increase in vaginal pH disrupts the suppressive role of the vaginal microbiota against additional pathogenic bacteria. Establishment by pathogenic bacteria triggers the secretion of various abnormal metabolites, such as sialidase, *β*-glucuronidase, coagulase, and neuraminidase, which diminish the thickness of the cervicovaginal fluid (CVF) and impair its protective role. In addition, mucosal immune and inflammatory responses are altered.

BV is a condition caused by multiple types of microorganisms and an exact microbiological definition remains elusive. It is featured by a decrease in *Lactobacilli* and an elevation in other bacteria typically associated with BV (Pramanick et al., 2019). Nevertheless, these causative organisms are capable of residing in the vaginal flora of healthy women. Meanwhile, individuals with BV typically do not show a notable inflammation. The vaginal mucosa is usually not inflamed or sensitive. Complaints of discomfort or pain are not often heard from female counterparts. Hence, BV is generally categorized as BV rather than vaginitis, and it’s regarded as a significant bacterial imbalance disorder. As depicted in Figure 2, in a healthy vagina during the probiotic phase, the lactic acid colony produces lactic acid. This lactic acid production helps maintain a vaginal pH below 4.5, establishing an acidic condition where acid-resistant bacteria can thrive while inhibiting the growth of pathogenic bacteria. Furthermore, lactic acid exhibits anti-inflammatory properties and serves to protect the epithelial cells. Additionally, it helps maintain the viscoelasticity of the mucus. In contrast, during dysbiosis and BV, there is a restriction in the population of *Lactobacilli*, leading to a reduction in lactic acid levels. This creates an environment where other pathogenic bacteria, such as *Prevotella, Gardnerella*, *Atopobium*, and *Mobiluncus*, can proliferate. These pathogens produce significant amounts of short-chain fatty acids (SCFAs) from glucose, maltose, and certain amino acids. SCFAs induces the activation of nodular lupus-like receptor protein 3 (NLRP3) inflammatory vesicles and IL-18 secretion in epithelial cells, promoting neutrophil recruitment to inflammatory sites. It also enhances the differentiation and suppressive function of FOXP3 + regulatory T (Treg) cells to mediate inflammation (Yu et al., 2025). In such instances, the integrity of the epithelium is compromised due to bacterial virulence factors, leading to impaired viscoelasticity of the mucus. This creates a pro-inflammatory environment characterized by a foul odor.

## 4 Association of vaginal microbiota with HPV infection and cervical cancer

### 4.1 HPV infection

The environment in which our bodies live is rich in microbiota, and the resident microbiota is considered to be an inseparable component of the human body. Research into the human microbiota has consistently shown its importance in preserving health and warding off a range of ailments, including gastrointestinal disorders and cancer. Research on vaginal microbiota and its functions is crucial because it is closely linked to various gynecological cancers (Liu et al., 2020). *Lactobacilli* in the healthy vaginal help immunocytes to clear HPV infections, prevent viral evasion, and reestablish immune balance. Certainly, dysbiosis of the vaginal flora has been linked to increased susceptibility to HPV infection (Nicolò et al., 2021; Cheng L. et al., 2020; Chao et al., 2019) and has been correlated with the persistence of HPV infection as well as the presence of HPV-associated lesions (Usyk et al., 2020; Brusselaers et al., 2019; Kwasniewski et al., 2018; Dareng et al., 2016; Audirac-Chalifour et al., 2016; Godoy-Vitorino et al., 2018). According to a meta-analysis conducted by Norenhag, it was found that having a vaginal microbiota dominated by non-*Lactobacilli* or even by *Lactobacilli* increased the chance of getting HPV significantly, by 3–5 times. Furthermore, research indicates that women who test positive for HPV tend to exhibit significantly higher microbial diversity in their vaginal microbiota and a decreased presence of *Lactobacilli* compared to those who test negative for HPV (Lee et al., 2013). Increased prevalence and incidence rates of HPV infection are closely associated with BV characterized by reduced *Lactobacilli* and increased anaerobic bacteria (Guo et al., 2012). The most prominent one is the increase of *Prevotella* bacteria associated with the recurrence of cervical cancer (Tsementzi et al., 2025). Additionally, genital infections such as *Chlamydia trachomatis*, *Mycoplasma genitalium*, and concurrent infections are associated with promoted HPV infection and hindered clearance of high-risk HPV (hr-HPV) strains (Cheng W. et al., 2020; Yang et al., 2020). Vaginal dysbiosis increases susceptibility to HPV infection. However, evidence suggests that transient HPV infection can reduce the richness and diversity of the vaginal microbiome, resulting in dysbiosis (Guo et al., 2025; Kumari and Bhor, 2021). Overall, HPV infection and vaginal dysbiosis appear to influence each other in a dynamic and reciprocal manner.

HPV infection is frequently encountered among sexually active women, with over 80% expected to acquire HPV at some phase of their life. While the majority can clear HPV infection naturally within 1–2 years, around 10–20% of women experience persistent HPV infection. The HPV virus is classified into two types based on its oncogenic potential: hr-HPV and low-risk (lr-HPV) types. The hr-HPV types include 16, 18, 31, 33, 35, 39, 45, 51, 52, 53, 56, 58, 59, 66, and 68, while the remaining types are classified as lr-HPV. The hr-HPV 16 and 18 types are detected in approximately 70% of cervical cancer cases. Continued HPV infection is considered a key factor in the development of cervical intraepithelial neoplasia and ultimately cervical cancer. Moreover, the lr-HPV can contribute to the development of genital warts, which can manifest as either benign growths or less aggressive lesions.

Furthermore, HPV impacts the maturation and differentiation of monocytes (Song et al., 2015), as well as inhibits the activity and role of TLRs, which play an essential role in recognizing pathogens (Daud et al., 2011). The expression of HPV-related tumor proteins (E6/E7) hamper the recruitment of macrophages along with other immune cells and suppresses the production of interferons (IFNs) by natural killer (NK) cells. IFNs are vital for curbing replication of the virus and activation of immune cells (Lee et al., 2001). In the realm of acquired immune responses, studies indicate that HPV 16 E6 and E7 diminish the expression of IFNs and human leukocyte antigen (HLA) (Tamadonfar et al., 2023) class I molecules, leading to a deficiency of cytotoxic T lymphocyte (CTL) feedback to HPV. Meanwhile, HPV controls the expression of a range of cytokines with anti-inflammatory and pro-inflammatory properties, influencing the roles of immune cells such as antigen-presenting cells (APCs) and CD4 + T cells (Cho et al., 2001). The ability of HPV to circumvent the immune response contributes to persistent infection and the advancement of cervical lesions. Acute HPV infection is characterized by the virus invading cells, viral genetic replication, and the production of virus particles, which may ultimately be eliminated by the immune system. Indeed, in certain instances of acute HPV infection, the virus is eliminated superficially, but the viral genome remains persistently within the infected cells and is difficult to detect, leading to a state of latent infection. Latent HPV infection has the potential to be reactivated by various factors, including immunosuppression. Chronic HPV infection is characterized as an untreated acute infection leading to sustained viral activity.

### 4.2 Cervical cancer

While most HPV infections are transient, a fraction persists and, over time, can initiate a carcinogenic cascade. The progression from persistent hr-HPV infection to cervical cancer is a multi-step process, and the vaginal microbiome is increasingly implicated in driving this progression. Cervical cancer stands as one of the most prevalent and deadliest cancers affecting women globally, with its morbidity and mortality rates on the rise. In the majority of patients, the immune system clears HPV infection within 6–18 months. However, when hr-HPV infection persists, it can lead to the development of cervical cancer. Multiple factors, including smoking, the amount and type of virus, age, as well as immune status, are strongly linked to the prolonged presence of HPV infection. The main treatment remains a combination of surgery procedure and chemotherapy. Certainly, long-lasting HPV infection and the resulting abnormalities in the cervix not only cause considerable physical distress but also place substantial psychological and economic strain on affected individuals. Indeed, previous research has indicated that the vaginal microenvironment plays a crucial role in shaping the progression of HPV infection and the development of cervical lesions. A dysregulated and functionally impaired microbiome tend to promote chronic low-grade inflammation, immune dysregulation, oxidative stress, DNA damage and metabolic rewiring, contributing to the carcinogenic effects of hr-HPV integrating oncogenes into the host genome. Vaginal inflammation increases the risk of cervical cancer (Huang et al., 2024). Lately, there has been growing attention to investigating the impact of vaginal microbiome dysbiosis on the persistence of HPV infection and the formation of cervical lesions (Qingqing et al., 2021; Santella et al., 2022).

Cervical cancer generally develops across four stages: the spread of HPV, persistent viral infection, the formation of cervical precancerous lesions within persistently infected epithelial cells, and eventual invasion of the epithelial basal membrane. Throughout the differentiation of basal cells, the HPV virus undergoes proliferation and sheds viral particles from the outer epithelial cells. Indeed, as HPV DNA becomes integrated into the host cell genome, it prompts elevated synthesis of E6 and E7 proteins. These viral tumor proteins play crucial roles in reactivating the synthesis of host cell DNA, fostering ongoing cell cycle processes, and inducing genetic changes, thus promoting the development of cancer. Moreover, HPV can alter glucose metabolism and promote glycolysis, creating a conducive microenvironment for cancer proliferation.

However, the immune system demonstrates the ability to clear most HPV infections, with only a minor fraction developing into precancerous or cervical lesions. Most patients either remain asymptomatic or exhibit transient positivity for the virus (Mosmann et al., 2021; Bhatla et al., 2013; Sangpichai et al., 2019). Certainly, recent findings affirm that the mere occurrence of HPV is insufficient to elucidate the onset and development of cervical cancer. Other factors, including host immune response, viral genotype, and environmental influences, likely play crucial roles in determining disease outcomes. There appears to be a consensus regarding the influence of immunity and hormones, but there is limited agreement on the role of the microbiota and changes in its composition. Some studies suggest a shift toward *Lactobacillus liners* dominance over *Lactobacillus crispatus*, while others indicate decreases in *Bifidobacterium bifidum*, *Enterobacter*, *Atopobium*, and *Streptococcus species* (Ochoa-Repáraz et al., 2017). Recent studies have firmly established that the vaginal microbiome plays a key role in the natural history of HPV infection and its development toward cervical tumors (Bautista et al., 2025). These changes have led to the belief that oncogenic mutations are a multistep process with multiple factors involved over time. This scenario is characterized by the fact that pathogen-infected cells are undergoing overlapping conditions of DNA alteration, which allows genetic and epigenetic alterations that enable viral replication and establish ideal conditions for oncogenic transformations (Ochoa-Repáraz et al., 2017; Hochreiter et al., 2000; Alagarasu et al., 2021; Paul and Grossman, 2014; Higgins et al., 2002; Collinson et al., 2002).

### 4.3 Mechanistic pathways linking vaginal microbiota, HPV persistence, and cervical carcinogenesis

#### 4.3.1 Microbial metabolic pathways

Research has demonstrated that alterations in the microbiome, instead of the influence of a solitary pathogen, are responsible for the majority of microbial carcinogenesis cases (Sobhani et al., 2019). A decline in *Lactobacilli* dominance decreases lactic acid production, disrupts pH homeostasis, and weakens the natural antimicrobial barrier. This favors the survival of anaerobes associated with HPV persistence. These anaerobes produce metabolites, including short-chain fatty acids, polyamines, ammonia, and volatile amines, that can irritate the epithelium, damage DNA, and cause oxidative stress.

These metabolic disturbances also interact with host cellular pathways. Increased levels of oxidative and nitrative metabolites promote lipid peroxidation, protein modification and genotoxic lesions. These processes impair epithelial defense and facilitate viral integration. Microbial enzymes that degrade mucins or generate reactive metabolites further compromise barrier function, thereby enhancing susceptibility to viral entry. Additionally, shifts in microbial metabolism can modulate host immune responses by altering cytokine profiles and pattern recognition receptors (PRRs) activation.

#### 4.3.2 Immunological interactions

The formation of tumors is closely associated with immune regulatory mechanisms within the vaginal epithelium. The vaginal epithelium of the host contains cell surface or intracellular receptors, known as PRRs, including TLRs and nucleotide-binding domain (NOD)-containing proteins. These receptors recognize and bind to pathogen-associated molecular patterns (PAMPs). In immune cells, TLR activation triggers a canonical signaling cascade beginning with the recruitment of the adaptor protein myeloid differentiation primary response 88 (MyD88). This process leads to the activation of the B-cell nuclear factor-κB (NF-κB) complex, which in turn promotes the transcription of cytokines and inflammatory genes (Crespo-Quiles and Femenía, 2025). Persistent HPV infection increases the expression of TLRs 2, 4 and 5 in the cytoplasm, leading to dysregulation of cytokine signaling. Inflammatory mediators such as IL-6, IL-8 and IL-1β promote tumor cell proliferation and drive cervical cancer progression (Schäfer et al., 2013). Beyond innate immune activation, HPV employs multiple immune-evasion strategies. A key mechanism is the downregulation of MHC-I molecules mediated by the viral protein E5, which retains MHC-I within the Golgi and endoplasmic reticulum, reducing antigen presentation to CD8^+^ T cells and facilitating persistent infection (Miyauchi et al., 2023).

Additionally, the abnormal activation of specific immune signaling cascades promotes carcinogenesis. The Janus kinase (JAK)/signal transducer and activator of transcription (STAT) pathway facilitates immune evasion, while STAT3 promotes the production of inhibitory cytokines, such as transforming growth factor beta (TGF-*β*), IL-6 and IL-10. This encourages the recruitment of regulatory T cells and inhibits dendritic cell maturation. Together, these changes create an immunosuppressive environment that encourages tumor growth.

**Figure 3:**
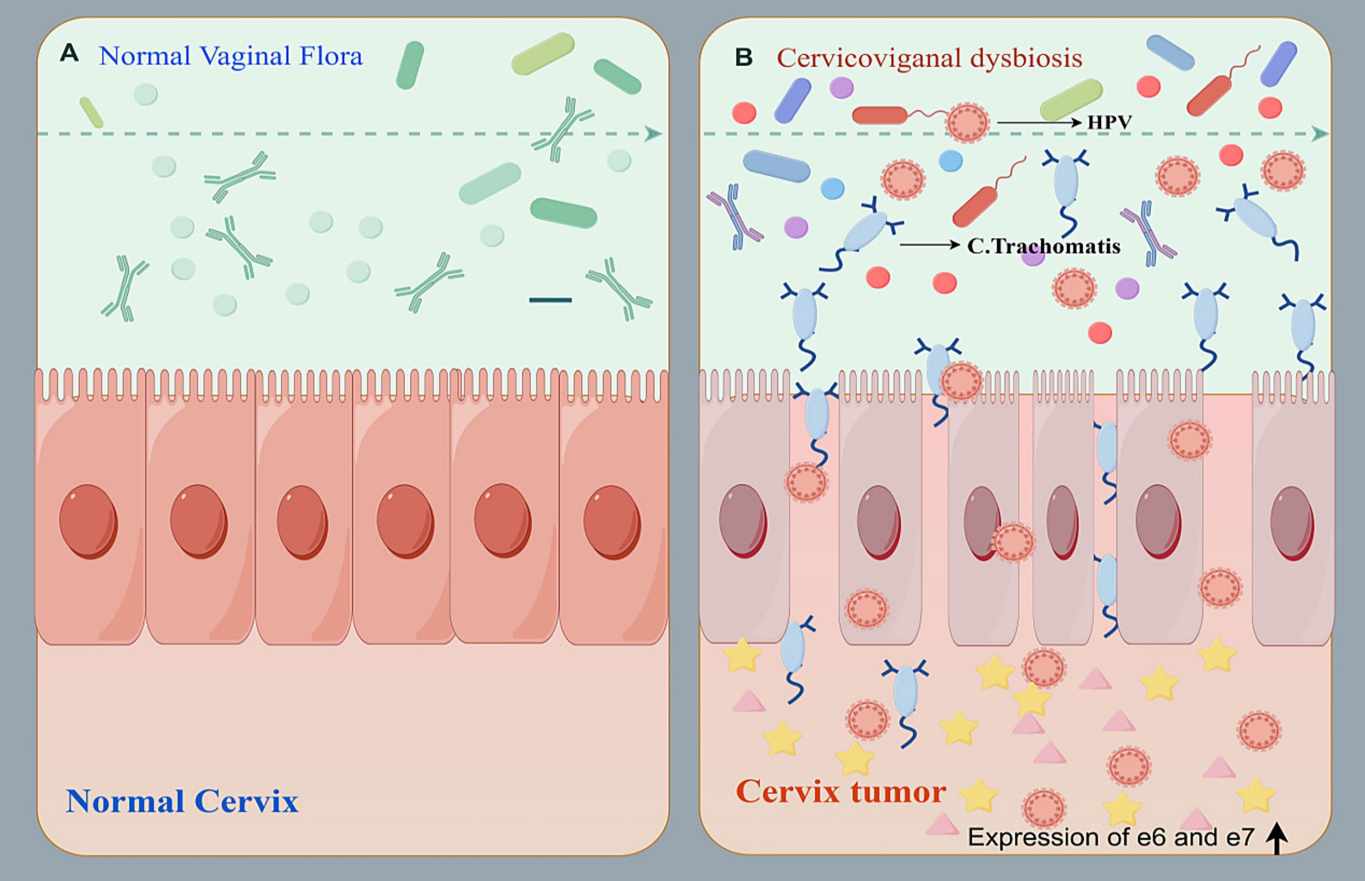
Role of cervicovaginal dysbiosis and co-infection in cervical tumor development. Cervicovaginal dysbiosis and HPV co-infection with other bacteria and viruses cause associated immune dysregulation leading to cervical tumors.

HPV further promotes cervical carcinogenesis through its E6 and E7 oncoproteins. E6 recruits E6AP to target p53 for proteasomal degradation, impairing apoptosis and DNA-damage responses. E7 binds and inactivates the retinoblastoma protein (pRb), releasing E2F transcription factors and driving uncontrolled cell-cycle progression. HPV E6/E7 oncoproteins also disrupt host immune surveillance by modulating transcription factor expression and perturbing NF-κB signaling (Zhang et al., 2025). This ultimately promotes abnormal cell proliferation, inflammation, angiogenesis, immune escape and tissue infiltration. Cervicovaginal dysbiosis is thought to exacerbate this process by potentially facilitating HPV acquisition and persistence, and promoting viral transformation (including E6/E7 expression, viral integration, and genomic instability), and supporting secondary infections, such as those caused by *Chlamydia trachomatis* (Figure 3). These immune disturbances together sustain a pro-inflammatory microenvironment that accelerates tumor formation.

#### 4.3.3 HPV–microbiota molecular mechanisms

There is increasing evidence that oxidative and nitric stress are key microbial factors that drive inflammation-mediated carcinogenesis (Nelson et al., 2015). Oxidative stress impairs the function of immune cells, while nitric stress increases the production of biogenic amines and nitrosamines, thereby strengthening the resilience of pathogens and supporting the formation of biofilms (Nelson et al., 2015). However, high concentrations of *Lactobacillus* species can counteract these effects by inhibiting amine-producing bacteria and exhibiting cytotoxicity toward cervical cancer cells.

At a molecular level, the interactions between HPV and the microbiome influence the expression of viral oncogenes and downstream tumor-promoting pathways. For example, the regulation of HPV16 E7 by miR-27b enhances the proliferation and invasion of cervical cancer cells (Zhang et al., 2015). Furthermore, E6/E7 activation stimulates the PD-1/PD-L1 axis, thereby driving tumor progression. Immune checkpoint inhibitors that target this pathway have shown promise as a treatment for metastatic cervical cancer.

#### 4.3.4 Barrier integrity and inflammation

Chronic HPV infection disrupts the normal cervical-vaginal microbiota and mucosal metabolism, triggering persistent inflammatory signalling. Activation of local mucosal immunity, including hyperactivity of NK cells and macrophages, promotes persistent tissue damage. However, HPV infection alone is not enough to cause malignant transformation. Additional factors, such as reduced *Lactobacilli* abundance, increased production of mucosal injury mediators and recurrent mixed microbial infections, all play a role in compromising epithelial barrier integrity.

Barrier dysfunction increases epithelial permeability, enabling inflammatory cytokines to penetrate deeper tissues and exacerbate intraepithelial damage. These processes promote HPV replication, transcription and modification, thereby advancing cervical intraepithelial neoplasia (Norenhag et al., 2020; So et al., 2020). Persistent inflammation further induces cytotoxic effects in healthy cells, causing DNA damage and activating autocrine and paracrine signalling loops that are influenced by epigenetic and microbial factors.

## 5 Interrelationships between the vaginal microbiota and the immune-endocrine system

During acute HPV infection, the initial immune response is driven by cells with antiviral and bactericidal properties, including mucosal NK cells, macrophages (M1 type during the acute phase and M2 type during the repair phase), and epithelial cells. Inflammatory patterns are infrequently observed due to the evolutionary development of molecular strategies by HPV, enabling them to evade both innate and adaptive immune responses. The HPV16 E7 oncoprotein suppresses TLR9 expression through the NF-κB signaling pathway, inducing an immunosuppressive state that impedes interferon production. This mechanism obstructs immune surveillance via cytokine responses, thereby enabling HPV to achieve immune escape (Mohammed et al., 2025). Infections typically exhibit a notable presence of CD4+/CD25 + regulatory T cells, activated T helper 2 (Th2) cells, and suppression of cytotoxic function, resulting in T cell anergy (Koskela et al., 2000).

The interplay between the vaginal microbiota and the host endocrine system is complex and bidirectional. The vaginal microbiota can metabolize and modulate the local availability of hormones. For instance, certain bacteria express enzymes like *β*-glucuronidase, which can deconjugate estrogen metabolites, potentially influencing the local hormonal milieu. Conversely, host sex hormones are pivotal regulators of the vaginal ecosystem. Estrogen, in particular, promotes the proliferation of vaginal epithelial cells and glycogen deposition. Animal studies provide insights into the potential depth of microbiota-endocrine interactions. Studies have shown that transferring microbiota from adult male mice to immature females results in shifts in hormone levels (notably elevated testosterone), as well as changes in the metabolome and autoantibody production. For example, one study found no significant differences in bacterial DNA sequencing results obtained from the caecal contents of 4-week-old and 10-13-week-old mice. However, they observed *α*-diversity discrepancies between the sexes in prepubertal mice. Sequencing of the 16S rRNA gene in the male microbiome, female, and neutered male mice revealed some “unusual” characteristics: the microbiota of female mice was very similar to that of castrated males, with comparable composition and androgen levels (Yurkovetskiy et al., 2013). Similarly, samples collected during pregnancy demonstrated that the composition of the vaginal microbiota tended to shift, mirroring changes in hormone levels and microbial communities. From pregnancy I to pregnancy III, an increase in the solidified bacterial phylum was observed, which is characteristic of the vaginal microbiome of normal women (Amin et al., 2023). On the other hand, during BV, vaginal microbiota dysbiosis has been defined by a variety of bacterial taxa from specific phyla, including *Fusarium*, *Actinobacteria*, *Pseudomonas*, and *Bacillus*-like phyla (Chaban et al., 2014). Likewise, the vaginal microbiota in premenopausal and perimenopausal women is primarily dominated by the fungal phylum, whereas in postmenopausal women, it is chiefly comprised of *Pseudomonas*, *Bacteroidetes*, and *Actinobacteria phyla* (Amin et al., 2023; Chaban et al., 2014).

It is well-known that there is an interaction between the immune system and the microbiome, involving hormone receptors, various immune mediators, stem cells, different subpopulations of leukocytes, plasma cells, and antibodies such as IgG, IgM, and IgA, along with interleukins and inflammatory proteins. *Lactobacilli* can directly influence the production of interleukins (IL-1*β*, IL-6, IL-8, IL-2, IL-10, and IL-17) and play a role in balancing inflammation and anti-inflammation. In terms of immunology, hormones play a pivotal role by actively controlling the synthesis of antimicrobial peptides (such as β-defensins) and cytokines that promote inflammation. These molecules are locally produced by epithelial cells, particularly to protect spermatozoa and prevent infections. Estrogen significantly influences the composition of microbial populations at different life stages. For example, before puberty, the vaginal microbiota is predominantly composed of anaerobic species from the *Enterobacteriaceae* and *Staphylococcaceae* families. During puberty, estrogen promotes the accumulation of glycogen in mature epithelial cells (Yurkovetskiy et al., 2013). The digestion of glycogen produces maltotriose and *α*-dextrin, which serve as essential nutrients for *Lactobacilli* and facilitate the production of lactic acid (Yurkovetskiy et al., 2013).

**Figure 4:**
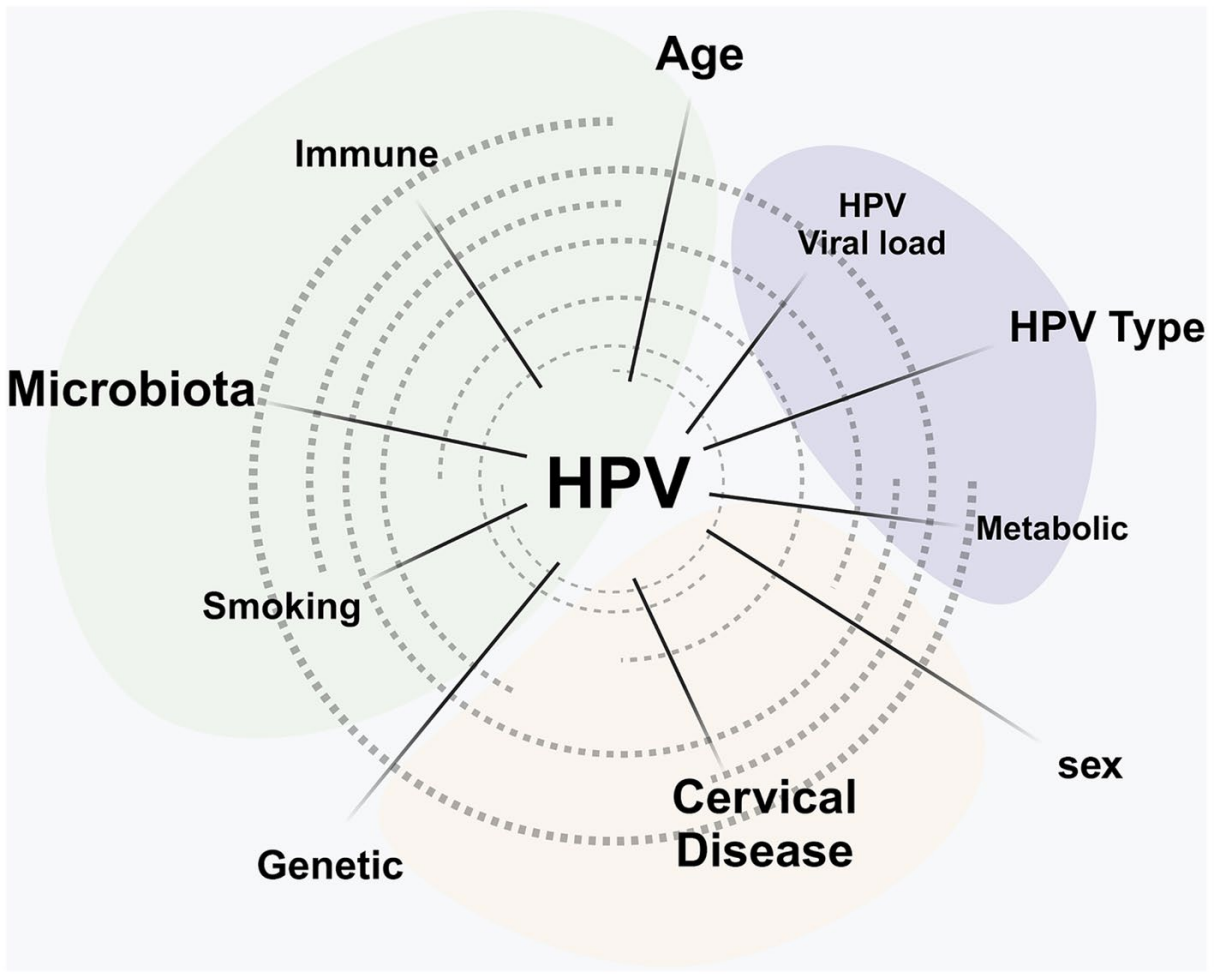
Factors affecting HPV progression. Host immunity, vaginal microbiota, viral characteristics, and lifestyle influence whether HPV infection is cleared, persists, or develops into precancerous lesions and cervical cancer.

As shown in Figure 4, these multifactorial influences highlight the complex interplay between host, viral, microbial, and environmental determinants in shaping cervical disease risk. The microbiota, immune, and endocrine systems form a uniquely specialized unit that ensures vital bodily functions and coordinates essential processes. The microbiota and the immune system collaborate to defend the body against harmful pathogens. Meanwhile, the microbiota and endocrine system ensure normal metabolic activities in organs by managing systemic balance.

## 6 Microbiome-based biomarkers and diagnostic technologies

Advances in sequencing and multi-omics technologies have enabled the identification of microbiome-based biomarkers to assist with the diagnosis and risk stratification of HPV persistence and cervical lesion progression, as well as predicting them. Technologies such as 16S rRNA sequencing, metagenomics, metatranscriptomics and metabolomics now enable high-resolution profiling of cervicovaginal communities and their functional outputs. This transformation turns the microbiome from a descriptive ecological feature into a clinically relevant diagnostic tool.

Microbial metabolites in the female reproductive system are the end products of metabolic reactions triggered by the interaction between nutrients and microbiota. These metabolites can alter the status of the host through multiple pathways by coordinating physiological functions and response mechanisms. They also serve as key regulatory factors in inflammation of the female reproductive tract, malignant tumors, and pregnancy (Ghartey et al., 2015; Mcmillan et al., 2015). Typical vaginal microbial metabolites, such as lactic acid, hydrogen peroxide, ethanol and SCFAs, are emerging as functional biomarkers that reflect the health of the cervix and vagina (Liu et al., 2022). Increased SCFAs in the female reproductive system lead to a mixed anaerobic flora and induce inflammation (Aldunate et al., 2015; Amabebe and Anumba, 2020). These mixed anaerobic flora include *Streptococcus, Bacteroides, Gardnerella, Prevotella, Mycoplasma, and Mobiluncus*, which are commonly found in female genital tract diseases such as BV and vulvovaginal candidiasis (VVC) (Aldunate et al., 2015, Ceccarani et al., 2019, Vitali et al., 2015). Anaerobes associated with BV can produce SCFAs in the female reproductive system through carbohydrate fermentation and amino acid catabolism. The most common SCFAs found in the female reproductive tract include propionate, acetate, isovalerate, isobutyrate, and n-butyrate (Dandona et al., 2004). In vaginal dysbiosis, acetate and propionate often reach elevated millimolar concentrations (10–50 mM), markedly higher than levels observed in *Lactobacillus*-dominant states. In addition, SCFAs signal through G-protein coupled receptors such as GPR41 and GPR43, which are expressed on cervical epithelial and immune cells (Ceccarani et al., 2019). Activation of these receptors modulates cytokine secretion, epithelial permeability, and neutrophil recruitment, suggesting a direct pathway by which dysbiotic metabolic profiles influence local immunity. High concentrations of acetate and butyrate have been shown to alter tight-junction proteins and histone acetylation patterns in cervical epithelial cells, potentially facilitating viral entry and supporting HPV persistence. Taken together, the composition of the microbiome and its metabolic outputs provide a rich source of biomarkers for the early diagnosis, prognosis and prediction of risk. Based on this concept, several microbial taxa and multi-omics signatures have been suggested as potential clinical biomarkers.

Changes in the vaginal microbiome can be used to screen for precancerous lesions of cervical cancer. Certain bacteria can serve as biomarkers to help us determine whether HPV infection can develop into cervical cancer. Traditional biomarkers include immune markers and HPV genotyping. Hr-HPV can integrate into the host genome and, through its multi-copy presence in cells, be released in the form of circulating tumor DNA (ctDNA). Therefore, ctHPV DNA is a potential biomarker for cervical cancer (Sivars et al., 2023). Type-specific total viral load may serve as a robust diagnostic indicator for High-grade squamous intraepithelial lesion (HSIL) in HPV types 16, 18, and 58 (Kim et al., 2020). However, these markers alone are insufficient for accurate risk stratification. Recently, increasing attention has been directed toward microbial biomarkers, particularly those from the gastrointestinal and vaginal microbiota, due to their close association with the pathogenesis of HPV infection and cervical lesions. Numerous studies have shown that alterations in vaginal microbiome diversity may serve as early indicators for the diagnosis and prevention of various HPV-related conditions. It has been reported that *Gardnerella vaginalis* can disrupt cervical-vaginal microbial homeostasis and may serve as a key biomarker for the progression of HPV infection to HSIL (Kudela et al., 2021). Likewise, other studies have indicated that *Streptococcus sanguinis*, *anaerobic cocci*, and *anaerobic digestible streptococci* may also function as potential biomarkers for HSIL (Mitra et al., 2015). Continued advancements in this field have the potential to improve current diagnostic frameworks.

In recent years, the vast amount of information and data about the microbiome has revolutionized the field of microbiology. Multi-omics approaches provide a more comprehensive understanding of the vaginal microbiome and its interactions with HPV. Metagenomics identifies the composition of microbes and their functional genes. Metatranscriptomics captures the expression of genes in microbes and hosts (Jiang et al., 2019). Metabolomics quantifies bioactive metabolites, such as SCFAs and lactate, that influence mucosal immunity. Each “omics” layer provides different, yet complementary, information. Combining them provides a more complete view of HPV-microbiome interactions. Recent studies illustrate the value of this approach. Multi-omics profiling has revealed that HPV-positive women exhibit increased microbial diversity and an enrichment of *Gardnerella*-related metabolic pathways. There are also shifts toward pro-inflammatory metabolites, such as acetate and propionate. Furthermore, metatranscriptomic analyzes have demonstrated that HPV infection alters the expression of host epithelial immune genes, including those in interferon-related pathways. Meanwhile, metabolomic signatures have been shown to distinguish cervical intraepithelial neoplasi (CIN)1 from high-grade CIN lesions with high accuracy. Together, these findings emphasize that multi-omics integration can reveal biomarkers of HPV persistence and identify molecular pathways linking dysbiosis to cervical carcinogenesis.

## 7 Treatment: probiotics and prebiotics

The World Health Organization (WHO) characterizes probiotics as live microorganisms that, when consumed in appropriate quantities, offer advantageous effects on the host’s health. Metronidazole and lincomycin are commonly used antibiotics to treat BV. The efficacy of antibiotics in treating bacterial vaginosis or antifungals in addressing VVC is not always optimal, and recurrence rates tend to be high. While antibiotics may effectively inhibit anaerobic overgrowth, they often fail to restore *Lactobacilli* to their optimal levels. Extended use of antibiotics is also linked to numerous adverse effects and an increased risk of bacterial resistance. Hence, with the growing recognition of the role of *Lactobacilli* in maintaining vaginal health, vaginal probiotics containing *Lactobacillus* strains have become increasingly popular as both preventive and therapeutic interventions for vaginal dysbiosis. Vaginal probiotics containing *Lactobacilli* have been extensively researched and developed for the treatment of BV and VVC. Pregnant women with VVC have shown promising efficacy and safety after using probiotics, significantly improving their quality of life. Furthermore, probiotics show promise in offering some potential benefits to individuals experiencing constipation (Ang et al., 2022a; Ang et al., 2022b). For pregnant patients experiencing recurrent *staphylococcal* infections, probiotic preparations are a promising therapeutic option if antifungal drugs are contraindicated. Besides their therapeutic benefits, long-term oral probiotics offer protection for healthy women as well (Reid et al., 2003). Most probiotics play a key role in restoring the composition of the vaginal microbiota (Van De Wijgert and Verwijs, 2020; Lagenaur et al., 2021).

**Figure 5:**
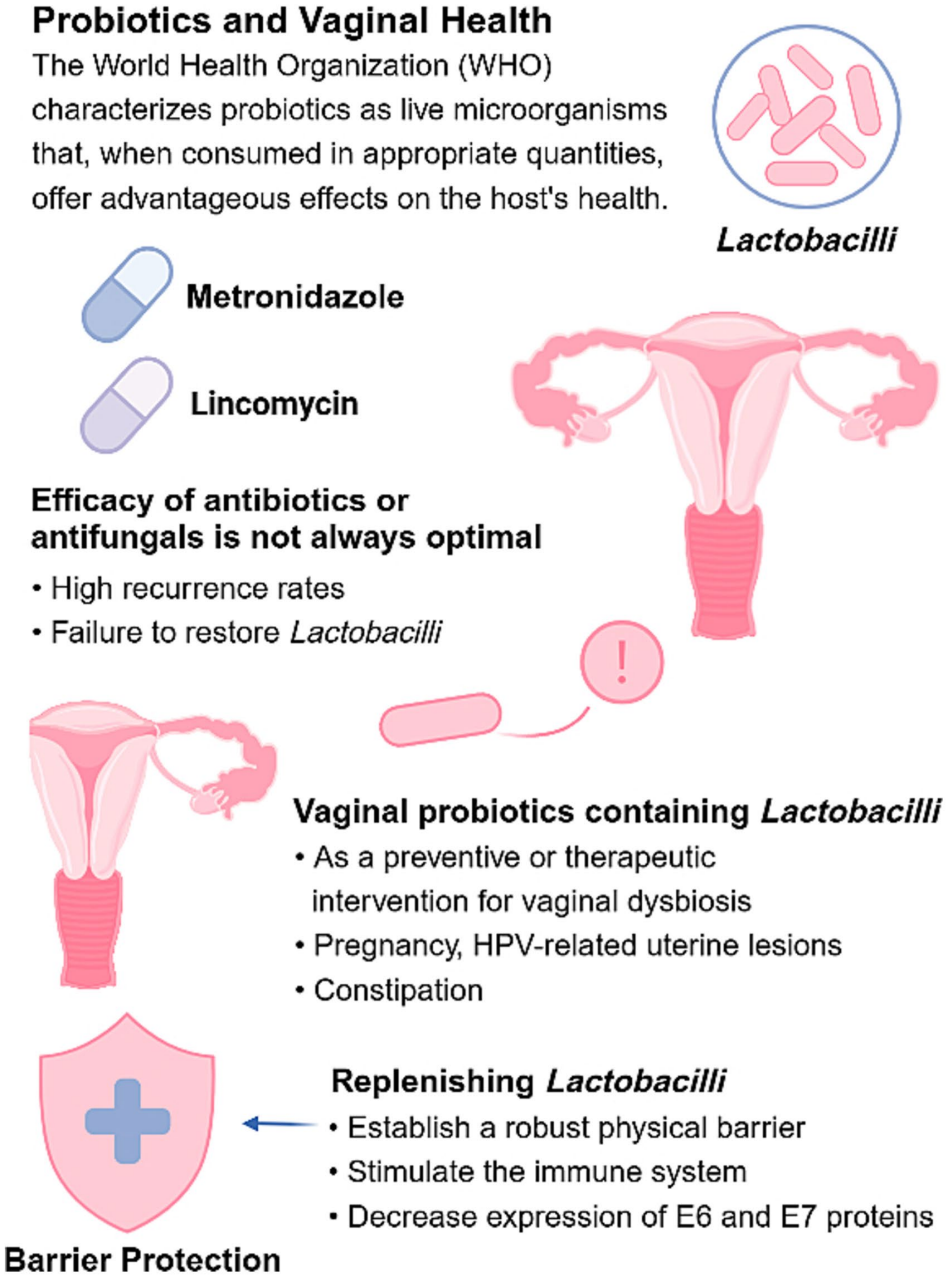
Probiotics and prebiotics in restoring the vaginal microbiota and reducing HPV-related risk. Probiotics help replenish beneficial *Lactobacilli* and support their growth. By improving the local microenvironment, these interventions may reduce HPV persistence and lower the risk of related cervical disease.

By replenishing diminished *Lactobacilli*, probiotics can offer protective functions comparable to natural *Lactobacilli*. These functions encompass establishing a robust physical barrier, stimulating the immune system, and decreasing the expression of E6 and E7 proteins. Studies have demonstrated that the *Lactobacillus strain LH23* can reduce the expression of E6 and E7 proteins, thereby inhibiting cervical cancer cell proliferation and inducing apoptosis (Hu et al., 2023). Additionally, recent findings indicate that taurine produced by *Lactobacillus rhamnosus* markedly decreases endometrial cancer cell viability, highlighting its targeted anticancer effects (Lawore et al., 2024). Our research findings indicate that women are more likely to develop vaginal dysbiosis in cases of persistent HPV infection. In this context, using probiotics might promote HPV clearance and is associated with a reduced incidence of uterine lesions in some studies (Medina-Rivero et al., 2025). Women experiencing persistent vaginal dysbiosis should consider using probiotics, reduce sexual frequency, and employ protective measures such as condom use to further mitigate the risk of complications (Figure 5).

Another method to restore vaginal microbiota is by using probiotics. Prebiotics are organic compounds that remain undigested or unabsorbed by the host, yet they selectively foster the metabolism and growth of beneficial bacteria within the body, consequently enhancing host health. Probiotics can enhance HPV clearance through immune regulation, competitive elimination of pathogens, and production of protective metabolites (Porcaro et al., 2025). We summarized some studies on the treatment of HPV infection and the prevention of cervical cancer with probiotics using Table 1. In cases of intestinal diseases, oral intake of prebiotics can improve intestinal conditions and promote the activity of *Lactobacilli*. Likewise, in the vaginal environment rich in *Lactobacilli*, prebiotics can exert comparable function. A large number of researchers have found that the disaccharide lactofructose broadly and specifically stimulates the growth of vaginal *Lactobacilli*, including *Bacteroides fragilis*, without promoting the growth of bacteria associated with bacterial vaginosis (Collins et al., 2018). Studies have additionally shown that probiotic maltose gel facilitates the transition of vaginal microbiota in rhesus monkeys from domination by BV-associated bacteria to a prevalence of *Lactobacilli* (Zhang et al., 2020). According to clinical studies, the combination of *Lactobacillus rhamnosus BMX54* with lactose can restore the vaginal microbiota and regulate vaginal pathological pathways (Baldacci et al., 2020). However, due to the lack of relevant clinical and preclinical studies, whether this combination can benefit patients with HPV infection needs to be further explored in the future. Despite these positive signals, the clinical evidence remains limited by several key factors: small sample sizes, heterogeneity in probiotic strains, dosages, and administration routes, short follow-up periods, and a primary focus on BV and VVC rather than HPV clearance as the primary endpoint. Consequently, while certain probiotic regimens show promise, they cannot yet be recommended as a standard clinical intervention for preventing or treating HPV-related cervical disease.

**Table 1:** Research on probiotics in the treatment of HPV infection and prevention of cervical cancer.

Probiotic strain	Experiment type	Dosage	Route of administration	Duration of probiotic consumption	Results	References
*Bifidobacterium adolescentis SPM1005-A*	*in vitro*	5.1 × 10^7^ CFU/mL (culture medium)		0–72 h	Suppression of E6 and E7 oncogenes	CHA et al., 2012
*Lacticaseibacillus casei LH23*	*in vitro*	1.25–5 mg/mL (bacterial lysates)		24–72 h	Suppression of proliferation and induction of apoptosis Suppression of E6 and E7 oncogenes metastasis were suppressed	HU et al., 2023
*Lactobacillus gasseri LGV03*	*in vitro*	1 × 10^7^ CFU/mL (culture medium)		48 h	*Lactobacilli* alter the host epithelial immune response in human ectocervical Ect1/E6E7 and cervical CaSki cells	GAO et al., 2023
*Lactobacillus crispatus chen-01*	*in vivo* (vaginal)	1 × 10^9^ CFU/capsule (lyophilized)	Vaginal Administration	3–5 months	decreases the diversity of vaginal microbiota	LIU et al., 2024
*Lactiplantibacillus plantarum Probio87*	*in vivo* (oral)	10^9^ CFU/sachet (lyophilized)	Oral Administration	12 weeks	Decreased Vaginal abundance of HPV	Xu et al., 2025
*Lactobacillus rhamnosus BMX 54*	*in vivo* (vaginal)	10^4^ CFU/tablet (vaginal tablet)	Vaginal Administration	5 months	Long-term probiotic use doubled the chance of resolving HPV-related cytological abnormalities compared to short-term use.	PALMA et al., 2018

## 8 Conclusion, limitations, and future directions

**Figure 6:**
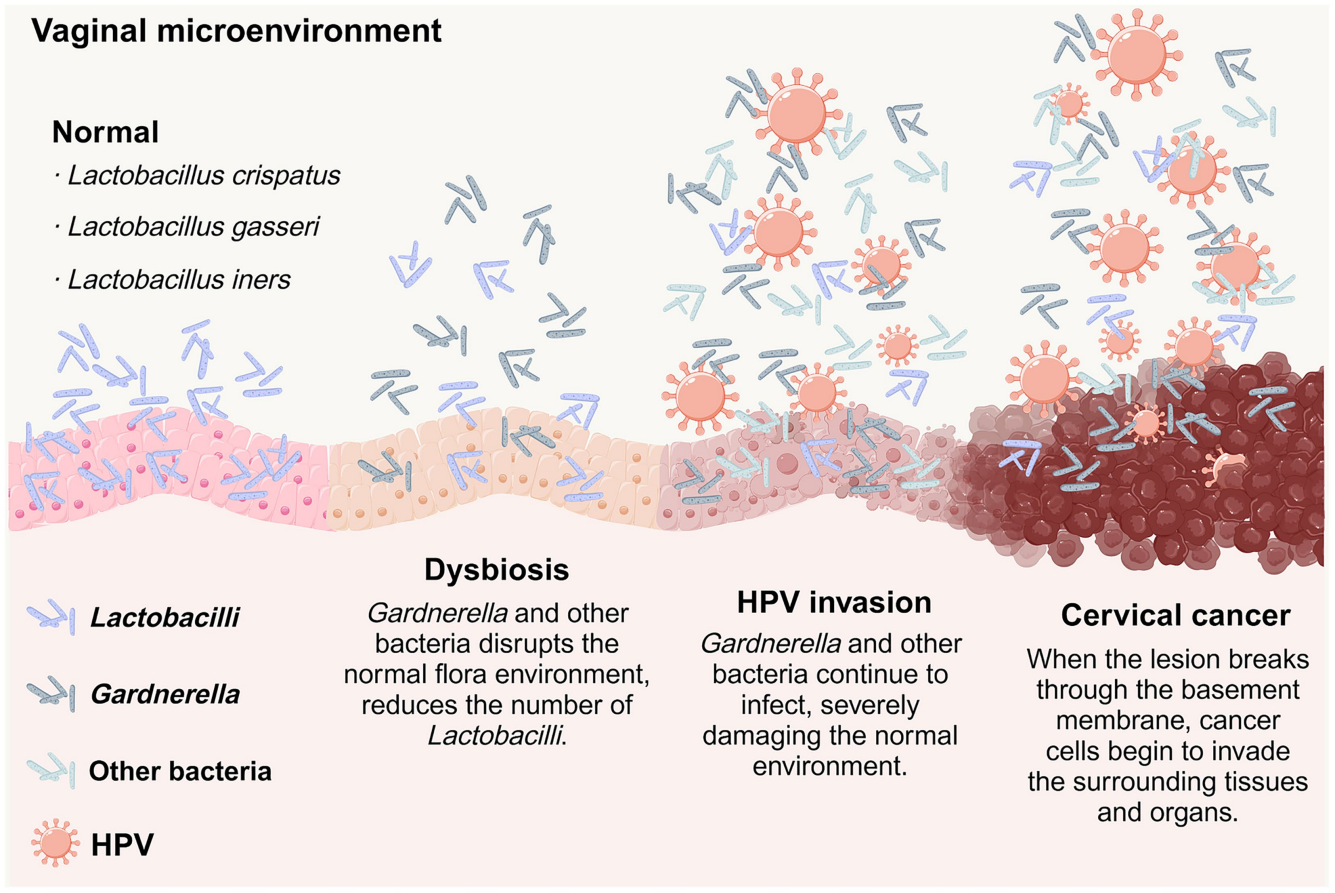
Progression of the vaginal microenvironment from homeostasis to dysbiosis, HPV invasion, and cancerous transformation. The vaginal environment shifts across four stages. Normal: The microbiota is dominated by *Lactobacilli*, which help protect the epithelium. Dysbiosis: Overgrowth of *Gardnerella* and other bacteria reduces *Lactobacilli* and disrupts the normal balance. HPV invasion: The damaged environment becomes more vulnerable to HPV infection, and continued microbial imbalance further harms the epithelium. Cervical cancer: With ongoing injury and viral persistence, lesions may progress and eventually invade beyond the basement membrane, leading to cancer development.

Numerous studies have shown that changes in microbial diversity and disturbances in microbiome composition can be linked to the onset of various diseases. Our assessment indicates that a reduction in *Lactobacilli* within the vaginal microbiota is linked to sustained HPV infection and the advancement of cervical lesions. The use of probiotics and prebiotics can aid in reestablishing the normal vaginal microbiota, thereby potentially impeding the progression of cervical lesions (Figure 6).

Despite extensive research on the relationship between the vaginal microbiome and HPV infection, most of the available data are derived from cross-sectional designs, which limit the scope for interpretation to correlations rather than causations. Additionally, inconsistent findings across studies are due to heterogeneity in sequencing platforms, CST classification methods and analytical pipelines. The dynamic and unstable nature of CSTs, coupled with hormonal fluctuations, sexual behavior and behavioral confounders, makes interpreting microbiome-HPV interactions even more challenging. These variables complicate the exclusion of external influences and often result in inconsistent or non-reproducible findings. However, it should be noted that it is unclear whether dysbiosis leads to persistent HPV infection or is a consequence of viral suppression of mucosal immunity. There is also insufficient mechanistic insight into how microbial metabolites (such as SCFAs) alter epithelial junctions and modulate inflammation. The intricate interplay between the microbiome, immune responses and HPV oncogene expression requires further investigation.

To address these challenges, future research should prioritize longitudinal cohort designs to clarify the temporal sequence between microbiome alterations and HPV infection. Integrating multi-omics platforms, which encompass metagenomics, metatranscriptomics, metabolomics and immunoassays, could help to identify the microbial metabolites and host pathways that drive HPV persistence or tumor formation. In future clinical trials, standardized microbiome-related markers such as SCFAs, lactate enantiomers and mucosal immune biomarkers could be used as secondary endpoints alongside HPV clearance rates. Well-designed, controlled clinical trials are needed to determine the therapeutic potential of probiotics, prebiotics, vaginal microbiota transplantation and metabolite-targeted therapies. Addressing these challenges will help future research clarify the clinical utility of microbiome modulation in preventing HPV persistence and cervical disease progression.
